# Book Review

**Published:** 1934

**Authors:** 


### Reviews of Books

**A Short History of Ophthalmology.** By A. Sorsby, M.D. Pp. vii., 103. Illustrated. London: John Bale, Sons and Danielsson, Ltd. 1933. Price 3s. 6d.—This very interesting little book contains the history of ophthalmology from about 2000 B.C. down to the end of last century. The introductory chapter deals with the dawn of ophthalmology in the ancient East—Babylon and Egypt. According to the Ebers papyrus (c. 1550 B.C.) the Egyptians knew such conditions as blepharitis, chalazion, iritis, cataract, and dacryocystitis, but almost the only surgical procedure it mentions is epilation. Hardly anything was known of the anatomy of the eye. The slow development of ophthalmology is traced in Greece, Alexandria and Rome. After the Mohammedan incursions ophthalmology seems to have taken a new lease of life. Hospitals, departmentalized very much in the modern manner, grew up and ophthalmic departments were always large and important. The chapters devoted to the anatomy, physiology and pathology of the eye show that it was not till the time of Leonardo da Vinci and Porta that the eye came to be regarded as a *camera obscura*. Mariotte discovered the "blind spot" in 1668. Porterfield showed, in 1759, that the blind spot was the entry of the optic nerve and that the retina was the essential organ of sight. The chapter on cataract contains a very good coloured illustration of the Indian operation of "couching"—the usual operation till Dairel published his account of the operation for extraction of the lens in 1748. Glaucoma has a chapter devoted to it. No definite conception of its nature existed till about 1840. In the chapter on therapeutics it is recorded that it was Augustin Prichard, of Bristol, who in 1851 resorted to excision of the eye to prevent sympathetic ophthalmia. There are chapters on spectacles, the ophthalmoscope and ophthalmology in the British Isles. The last gives a good deal of information about the notorious quack oculists and charlatans of the seventeenth and eighteenth centuries. There is a bibliography of the history of ophthalmology, and there are indexes of names and subjects.

**Surgical Anatomy and Physiology.** By N. C. LAKE, M.D., M.S., D.Sc., and C. J. MARSHALL, M.D., M.S. Pp. ix., 888. Illustrated. London: H. K. Lewis & Co. Ltd. 1934. Price 30s.—A text-book of nearly 900 pages is a formidable addition to the medical student's library, and this volume will probably find its place rather as a work of reference than as the daily companion of the embryo surgeon. In this capacity it is truly excellent, although the emphasis is distinctly on anatomy rather than surgery or physiology. The diagrams are clear and well chosen, and include, beside many old friends, a number of skiagrams that are particularly valuable from the clinical side.

**Surgical Applied Anatomy.** By SIR FREDERICK TREVES, Bart. Ninth edition. Revised by C. C. CHOYCE, M.D. Pp. x., 720. Illustrated. London: Cassell & Co. Ltd. 1934. Price 14s.—The appearance of a ninth edition of a work of this kind just fifty-one years after the first is, in itself, sufficient testimony that the book in question has filled an important place in the student's library for nearly two whole generations. This fact is not to be wondered at, for to many thousands of readers "Treves" has long been recognized as an old friend. The study of Applied Anatomy is not too popular with students, and a readable book on what is really a most important subject is badly needed. This book is essentially readable, and that is one reason why it has retained its popularity for so long a period of time. The author of this edition has departed from what he calls Treves's "anecdotal" style, and although in some ways that is to be regretted, the decision on the whole is probably a wise one. It may be said that the whole work has been thoroughly and competently revised, and that is nearly all that need be said. In its new guise it will continue faithfully to serve the present generation as it has an earlier one. A careful search reveals nothing omitted that might usefully have been included, and only one mistake—a misprint in the fourth line of page 468.

**A Synopsis of Medicine.** By H. L. TIDY, M.D. Sixth Edition. Pp. xvi., 1,112. Bristol: John Wright & Sons Ltd. 1934. Price 21s.—The sixth edition of Tidy's *Synopsis* is an additional proof that this book is very widely appreciated. The advances made in the four years since the fifth edition was published have made extensive revision necessary. This has been very well carried out. The descriptions given of the disorders resulting from disturbances of the parathyroid functions deserve the highest praise. So too does the chapter on the anæmias. Additions to our knowledge of the blood and its disorders are being made so rapidly that the difficulties of differentiating and classifying the anæmias are immense, but the author's plan offers a very solid foundation from which to follow future progress. There is no need to enumerate all the new matter. A glance at the preface will satisfy most of our readers of the necessity of getting the new edition.

**Industrial Maladies.** By SIR THOMAS LEGGE, M.D. Pp. xvi., 234. Illustrated. London: Oxford University Press. 1934. Price 12s. 6d.—Workmen's compensation is very definitely on the increase, and more and more cases are seen by both general practitioners and specialists, which demand not only a knowledge of industrial maladies, but of the processes and agents by which they are brought about. This book deserves, therefore, to be widely read, and will be particularly appreciated by certifying surgeons. The preface contains a life history and an account of the great work of Sir Thomas Legge. The subject-matter is well presented in fifteen chapters, and deals with industrial poisoning by lead, phosphorus, mercury, arsenic, and other metallic compounds. Dermatitis, pulmonary diseases due to dust, and tar cancer are thoroughly considered. There are thirteen good photographic plates illustrating these. The author does not introduce the more surgical conditions, hence there is no account of bursitis, or injuries to the back, and like conditions. The book makes interesting reading, as it contains historical notes on several diseases—a good account of glass blowers' cataract is given. The graphs are disappointing and are difficult to read, and a better method of presenting graph 9 might be found. Not everyone will agree with some of the views expressed, *e.g.* page 51: "There is a fairly close relation between the degree of exposure to lead and the proportion found in the urine and the medical condition of the exposed persons." This has been found on very many occasions not to be true. The index is good, and the book should prove a valuable one for reference. The work of such an eminent authority is worthy of addition to the libraries of all those more directly concerned with industrial diseases.

**Diffuse Sclerosis.** By L. BOUMAN, M.D. Pp. iv., 160. Illustrated. Bristol: John Wright & Sons Ltd. 1934. Price 15s.—This book summarizes very completely our present knowledge of a condition which under various synonyms, to wit, Schilder's disease and encephalitis periaxialis diffusa, has attracted a good deal of attention of late. The disease owes its interest to its relationship with other diseases exhibiting demyelinization of the nervous system, notably disseminated sclerosis and subacute combined degeneration. Regarded as a rarity, one wonders how often it passes unrecognized. Most of the literature on the subject has been scattered about in monographs. Professor Bouman's book puts the subject in a very readable form. It is excellently illustrated and contains a very complete bibliography. After a full discussion of the pathological anatomy and pathophysiology he summarizes the whole, indicating the difficulties of, and cardinal points in, diagnosis. He gives a detailed account of a number of personal cases which have been carefully studied throughout their illness to autopsy, together with excellent microphotographs of sections showing the specific lesions. The correlation of lesions and clinical signs is carefully discussed. While the book will primarily appeal to the neurologist, it should also be of considerable interest to the general reader. Without doubt, as a book of reference, it fulfils a very useful rôle.

**Clinical Electrocardiography.** By W. EVANS, M.D. Pp. x., 49. Illustrated. London : H. K. Lewis & Co. Ltd. 1934. Price 5s.—It is perhaps hardly an exaggeration to state that to many medical students and practitioners the electrocardiogram and its interpretation is shrouded in mystery, and rather savours of " black magic." This is perhaps because the majority of books dealing with the subject take the reader fairly deeply into the physics of the electrical reactions of contracting muscle. In this handbook, however, Dr. Evans, after a short introduction and a description of a " plan " to be followed in investigating a tracing and the main points to be noted, allows the actual electrocardiograms (of which there are sixty-four) to speak for themselves with the help of short explanatory notes. The book concludes with a series of " test " tracings, the key to which is given on the last pages. The electrocardiograms are perfect examples of the various abnormalities which they illustrate. Their technical quality is that which we have learnt to expect from the cardiac department of the London Hospital, *i.e.* beyond criticism. The book is well produced, and should do much to dispel the illusion that the interpretation of electrocardiograms is a mystic rite.

**Diseases of Infancy and Childhood.** By LEONARD G. PARSONS, M.D., F.R.C.P., and SEYMOUR BARLING, C.M.G., F.R.C.S. 2 vols. Pp. xxxviii., 1,798. Illustrated. London: Oxford University Press. 1933. Price £4 4s.—An apology is due to the editors and publishers of this treatise. Our review has been unduly delayed by the indisposition of the reviewer. Nevertheless this delay has not been entirely a disadvantage. It enables the reviewer to realize that Parsons and Barling has established a singular pre-eminence amongst books on children's diseases. The authors of the various articles have been well chosen and are all abreast of modern work and thought. It is impossible to do justice to the many good points in the book by quotation. A limited number of appreciative observations must suffice. The idea of opening the book with a chapter on heredity is sound as well as novel, and Dr. Cockayne has managed to compress an intricate subject clearly and intelligibly. The chapters on feeding and nutrition are first-rate, though the use of negro children to illustrate rickets was at first puzzling until we realized that the authors of these sections are working in the great school of Johns Hopkins. Maitland-Jones has contributed several sections, but that on infantile atrophy is outstanding. The acute exanthemata are well described, and one point is deserving of notice, as it is not widely recognized, namely that the administration of scarlatinal anti-toxin prevents or limits desquamation after scarlet fever. Ignorance of this fact sometimes leads to the original diagnosis being questioned. The duration of diphtheria immunity after protective inoculation is not clearly enough stated. In the bibliography to the chapter on tuberculosis Calmette's classic work *L'infection bacillaire et la Tuberculose* is not included, although in the text some mention is made of his *B.C.G.* E. Watson-Williams has written excellently on the diseases of the upper respiratory passages and of the ear. The colour illustrations of the appearances of the *membrana tympani* in various diseased conditions are both artistic and instructive. Reginald Miller has contributed the best modern account of rheumatic fever that we have seen. Lobar pneumonia is well described by Professor A. E. Naish, but it is verging on pedantry to intitle the chapter alveolar pneumonia. K. D. Wilkinson is good on heart disease and particularly lucid on congenital defects of the heart. If we have criticisms to make they are few, but the discussion of the recognition of the pre-paralytic stage of acute anterior polio-myelitis is inadequate and the treatment of the disease is very poor indeed. The aid of a surgeon was needed here to write about the modern treatment. A singular relic is included in the after-treatment of empyema: "bottle-blowing" is recommended. In these days of radioscopy have the "bottle-blowers" ever seen on the screen what happens to the unaffected lung? The two volumes are produced and illustrated with the care and excellence that characterize all the Oxford medical publications.

**The Clinical Study and Treatment of Sick Children.** By JOHN THOMSON, M.D., LL.D., and LEONARD FINDLAY, M.D., D.Sc. Fifth Edition. Pp. xxxvi., 1,075. Illustrated. Edinburgh: Oliver & Boyd. 1933. Price 30s.—The fifth edition of this standard text-book on pediatrics has been rewritten and enlarged by Dr. Leonard Findlay. Its position as one of the leading text-books on the subject in the English language was established long ago, and Dr. Findlay is to be congratulated on having fully maintained the reputation of his master's book by this revision. It might possibly have been better to have discarded the division of anæmias into primary and secondary, a distinction which nowadays is almost meaningless. The discussion of rheumatic fever is frankly disappointing. Surely in these days the disease is looked upon as a carditis with a tendency to arthritis, or to chorea or the appearance of sub-cutaneous nodules. The relegation of carditis to the third place in the "chief manifestations" seems old-fashioned. The chapter on congenital syphilis is admirably written and illustrated, though amongst the "traces of past syphilitic disease" the characteristic skin scars of the "Great Pox" are not mentioned, save in the form of scarring after phagedænic ulceration, which is uncommon. Infantile paralysis is not very well described as regards the pre-paralytic stage. The "tripod sign of Amos" or its modification as described by Dr. Macnamara are not given the prominence they deserve. Too much reliance is placed on the value of convalescent serum and too little importance is attached to the need for calling in an orthopædic surgeon as soon as the diagnosis of polio-myelitis is made. The chapters on feeding and nutrition are splendid reading, although the retention of Finkelstein's terms "bilanzstörung" and "dekomposition" in their German form was surely unnecessary. Some compact translation is not unattainable. But, take it all in all, "Thomson & Findlay" is a rare good book.

**What to do in Cases of Poisoning.** By W. MURRELL, Fourteenth Edition. Pp. vi., 208. London: H. K. Lewis & Co. Ltd. 1934. Price 5s.—It can be safely said that any publication which has reached, as this one has, its fourteenth edition, has found a public, and so is already assured of success. This last edition has been revised, and new poisons have been added. The chapter on antimony poisoning from enamel ware glaze should prove especially interesting in view of the controversy that it has recently raised. As those who have this book will know, the poisons with their symptoms and treatment are set out in alphabetical order, and that together with an elaborate index will make it especially useful in an emergency. No general practitioner should be without this in his desk or on his bookshelf.

**High Blood Pressure.** By J. F. HALLS DALLY, M.D., M.R.C.P. Third Edition. Pp. 281. Illustrated. London: William Heinemann Ltd. Price 15s. This third edition of *High Blood Pressure*, by Dr. Halls Dally, has been very largely rewritten and many new illustrations have been added. The chapters on sphygmomanometric methods and instruments are particularly valuable. Standard arterial pressures at various ages are discussed in the light of recent knowledge, with the result that these standards are now lowered considerably. The fallacious standard that the proper adult systolic pressure is the age *plus* one hundred finds no support. Dr. Halls Dally gives no normal systolic pressure as being above 150 m.m. at any age. Halls Dally on high blood pressure becomes, with this third edition, an absolutely indispensable book for every practitioner of medicine, and for none more than those engaged in medical examination for life assurance.

**The Etiology and Treatment of Spasmodic Bronchial Asthma.** By H. G. OLIVER, M.D. Pp. vii., 48. Illustrated. London: H. K. Lewis & Co. Ltd. 1934. Price 3s. 6d.—This little volume is devoted to propounding the thesis that spasmodic asthma is due to a mycotic infection, a monilia being suggested as the causal organism. Those who have had experience in the field of mycoses will realize that such a thesis needs the closest criticism, and the most unmistakable experimental demonstration. Dr. Oliver's "proofs" are so far based only on cultural recognition of the monilia in the sputum of asthmatics.

**Diseases of the Skin.** By S. E. DORE, M.D., and J. L. FRANKLIN, M.D. Pp. xii., 410. Illustrated. London: Cassell & Co. Ltd. 1934. Price 10s. 6d.—This book provides a remarkably complete text-book of diseases of the skin, and will be welcomed by students and practitioners. Chapters on the structure and functions of the skin and the principles of diagnosis are followed by descriptions of the various diseases, the clinical appearance, ætiology, differential diagnosis, prognosis and treatment of each condition being given. A useful feature is the use of small type for the rarer conditions. There are no coloured plates, but forty-six black and white illustrations are included, all of these being photographs of actual cases. The only criticism which one has to make is that in many cases too little space is devoted to the treatment of the more common conditions, and it is hoped that this fault will be remedied in future editions.

**Melancholia in Everyday Practice.** By E. L. HOPEWELL-ASH, M.D. Pp. vi., 136. Illustrated. London: John Bale, Sons & Danielsson Ltd. 1934. Price 7s. 6d.—This book will have served a useful purpose if it enables the general practitioner to recognize those cases of melancholia in which the danger of suicide is certain. Its perusal may in consequence save the lives of some useful members of society among their patients who have fallen victims to morbid depression. In giving a careful review of various cases of manic-depressive mental disorder and involutional melancholia, the author demonstrates the importance of mild cases of melancholia that are often missed or diagnosed as physical illnesses. The chapter on treatment is good, but the author has not appreciated the need for carefully graduated occupational therapy prescribed by the doctor. The author rightly insists on compulsory restraint for every patient who threatens suicide, who refuses food or is dangerous, but it is much to be regretted that he insists on certification also. As a large proportion are willing to be treated as voluntary patients under the Board of Control, and temporary orders are available for all who have lost volition, only those few who refuse to be treated require certification.

**Atlas of the Commoner Skin Diseases.** By H. C. G. SEMON, M.D. Pp. 220. 103 coloured plates. Bristol: John Wright & Sons, Ltd. 1934. Price 42s.—This is a book which moves the reviewer to write with real enthusiasm. It presents us with over 100 most beautiful plates, shown by coloured photography, illustrating all the common skin diseases. The practitioner who keeps this by him is likely to derive the greatest possible help therefrom in the diagnosis of skin cases in his practice, a class of case that, it must be confessed, the majority of doctors find very difficult. There is a brief description of each condition illustrated, mentioning how such cases may be diagnosed and treated. The author, photographer, and publishers must share in the credit for a truly beautiful piece of work.

**Head Injuries.** By L. BATHE RAWLING, M.B. Pp. viii., 86. Illustrated. London: Oxford University Press. 1934. Price 7s. 6d.—This monograph on head injuries by an acknowledged master on the subject is of extreme value to the practitioner in an age when the motor-car provides a regular supply of suitable subjects. The question of diagnosis is dealt with in a most practical way, and the pathology is well illustrated by means of illustrations of injured skulls. Treatment of each of the conditions described is adequately discussed, and the various pitfalls that may await the practitioner receive due notice. Altogether this is a very valuable contribution to our knowledge of the subject, and will find its place on the shelves of all progressive surgeons.

**L'Opération de Bassini.** By PROF. A. CATTERINA (Geneva). Pp. 57. Illustrated. Paris: Félix Alcan. 1934.—This is a monograph on Bassini's operation for the radical cure of inguinal hernia. Professor Catterina emphasizes the advantages of the original operation as described by Bassini himself over the usual modern variations of it. He describes in detail the technique observed by his master in the treatment of each of the layers encountered in the operation. Each step is given a separate paragraph and is illustrated by excellent paintings by Dr. Orazio Gaigher, who is himself a medical man. There are three points in which the operation described varies from the usual English text-book description: one is the removal of the cremasteric fascia; secondly, the definition of the free border of Poupart's ligament; and thirdly, the care taken to incise the transversalis fascia. The free border of the Poupart's ligament is the point to which the interrupted sutures in the conjoint tendon are fixed. The author claims that the careful technique that he has described, if it is followed strictly, should give a higher degree of security from recurrence than appears to be the experience at present of those who use the present modifications of Bassini's operation. At the end of the book a very complete list of complications associated with inguinal hernia and their treatment is given, each complication being treated by a concise annotation.

**Physical Signs in Clinical Surgery.** Fourth Edition. By Hamilton Bailey, F.R.C.S. Pp. xx., 287. Illustrated. Bristol: John Wright & Sons, Ltd. 1933. Price 21s.—The moment the reader opens this book he will appreciate the reasons for its popularity, which has been so great that in seven years no fewer than four editions have appeared. It consists essentially of a series of excellent surgical tutorial lectures, admirably illustrated by numerous and well-chosen pictures, the value of careful and systematic clinical examination being the theme. It is difficult to find words adequately to express the admiration that this work elicits, and the practitioner who does not possess it either has nothing to learn about surgery or has no desire to improve his professional equipment.

**Diseases of Women by Ten Teachers.** Edited by Comyns Berkeley, M.D., M.C. Fifth Edition. Pp. xii., 568. Illustrated. London: Edward Arnold & Co. 1934. Price 18s.—The fifth edition of *Ten Teachers* brings this sound and reliable work up to date. Its plural authorship ensures a balanced opinion on difficult and controversial points, and informs the reader of the current teaching in the various London schools. There is a valuable section on the endocrinology of the female, in which are set out the constantly changing opinions of physiologists and biochemists on this subject. The chapters on ovarian tumours contain some notable additions. Short accounts are also given of the use of diathermy, X-rays, and radium in the treatment of gynæcological conditions, and the new edition can be confidently recommended to students and practitioners alike.

**Aids to Obstetrics.** By L. Williams, M.D., M.S. Tenth Edition. Pp. viii., 219. London: Baillière, Tindall & Cox. 1934. Price 3s. 6d.—This little book is by no means a dull tabulation of the principal facts of obstetrics. In a brisk style, and without the use of telegraphic English, it presents the chief points of the subject, explains difficulties, and also gives several very useful practical hints. Used in conjunction with the larger text-books it should prove of great value to the student.

**Handbook of Filterable Viruses.** By R. W. FAIRBROTHER, M.D. Pp. ix., 193. London : William Heinemann Ltd. 1934. Price 7s. 6d.—The author of this handbook, well known in this country for his work on the viruses of poliomyelitis, is to be congratulated on having written a concise, readable account of the filterable viruses, in which all important work on this intricate subject is discussed and analysed with commendable impartiality. Technical details are omitted, but this omission makes for a clearer exposition of general principles, and there is no work of this size in which the general reader will find the subject so carefully reviewed.

**The B.C.G. Vaccine.** By K. N. IRVINE, D.M. Pp. ix., 70. London : Oxford University Press. 1934. Price 5s. — Dr. Neville Irvine's book on B.C.G. Vaccine is not entitled to rank as an authoritative criticism of the value of B.C.G., although it provides interesting grounds for reflecting on the superb indifference which British medicine has shown towards Calmette's work. Dr. Irvine has seen the administration of B.C.G. in New York and has interviewed some Hungarian workers. Chapter 3 indicates that he visited the Institut Pasteur in Paris during Professor Calmette's lifetime, but his visits do not seem to have been very informing. He hints at some doubts about the meaning of the instructions issued from the Institut, but his doubts read as if a few minutes' conversation would have cleared them up. The general impression is that Dr. Irvine's book tends to belittle by faint praise the wonderful efforts of the late Professor Calmette to grapple with the so far unsolved problem of how to protect children against the risk of infection with tuberculosis. We are told : " Though the favourable results in no way exonerate those investigators who first gave the vaccine to children, yet it must be admitted that sufficient work has now been done to prove that B.C.G. vaccine is harmless to the normal infant." Again : " A considerable amount of misunderstanding and opposition might have been avoided if Calmette would state that the B.C.G. is a ' virus fixe ' when cultured according to his instructions." This is just what Calmette personally told the reviewer, when he was invited by Calmette to go over to Dublin and try to induce the Irish Free State President to make a public trial of his B.C.G. vaccine. Having known Calmette for many years, one cannot praise his great energy and perseverance enough. He fought to save the lives of children, and the future will give him his right place among the great ones of this world. Calmette was always hurt at the neglect of his work by English doctors. The prevention of tuberculosis seems to be neglected in the country, and what is now needed is an investigation by our Ministry of Health, or some other public body, into the value of B.C.G., and any other means for protecting children against the risks of infection with tuberculosis. The issue of this book by the Oxford Medical Publications may help to stimulate an intelligent interest in B.C.G. amongst medical men in this country, and for that reason it is warmly welcome.

**Materia Medica for Nurses.** By A. M. CRAWFORD, M.D. Third Edition. Pp. viii., 100. London: H. K. Lewis & Co. Ltd. 1934. Price 3s. 6d.—This useful little book has now reached its third edition. Its general arrangement has not been altered, except for the introduction of two new chapters, one of which deals with the interpretation of prescriptions. This contains some information of no importance to nurses, *e.g. fiat emulsio*, or *haustus*, or *unguentum* being mere directions to the dispenser; whilst the common Latin abbreviations for "four-hourly," "six-hourly" or the initials "s.o.s." are omitted. No patient will come to harm because the nurse does not know the meaning of *cum* or *aqua bulliens*, but she ought to recognize "q.q.h." Why does Professor Crawford follow *in* with a nominative (or is it an accusative plural) *bis in dies?* The other new chapter consists of brief descriptions of commonly used drugs, including some proprietary preparations. These are badly placed, they should not have been separated from the groups to which they belong, *e.g. liquorice* should have been added to the chapter on vegetable purgatives and *nepenthe* to the opium and morphine chapter. This is a point for which perhaps the publishers and not the author are responsible.

**Garrod, Batten and Thursfield's Diseases of Children.** By H. THURSFIELD, D.M., F.R.C.P., and D. PATERSON, M.D., F.R.C.P. Third Edition. Pp. 1152. Illustrated. London: Edward Arnold & Co. 1934. Price 50s.—This excellent publication comes out in a third edition with a good many changes and additions to the text and in the illustrations. It has been brought thoroughly up to date and offers a sound and for the most part an eminently readable account of the best modern practice of pædiatrics. It is obviously impossible in a short review to do more than briefly to indicate the outstanding features of a work of 1,200 closely (but clearly) printed pages compiled by thirty-six eminent authors, each of them an acknowledged authority upon the subject treated of by him. But the practitioner and senior student will here find in nearly every case all that he can reasonably desire to know of the ætiology, pathology, symptomatology, and treatment of any medical disease in children which he may encounter. As a matter of fact, the scope of the work is much more ambitious than an attempt to deal merely with medical diseases of children. There are also chapters on orthopædics, on diseases of bone and joints, and on surgical diseases of other organs, such as the genito-urinary system. Sir H. D. Gillies writes on hare lip and cleft palate—indeed, a wide field of surgery is covered. These chapters on surgical affections and operative procedures are, perhaps, somewhat less satisfactory than the more medical side of the work. It is a task of great difficulty to compress orthopædic surgery, even in broad outline, into eight pages, and there seems to be, sometimes, a lack of balance in the treatment allotted to other diseases. For instance, cryptorchidism, which every practitioner encounters so frequently, receives but twelve lines, with never a hint of any treatment, whereas congenital valvular obstruction of the urethra, surely one of the rarer abnormalities, receives thirty, with an illustration. But these are only minor criticisms, and for the vast majority of the articles one can have nothing but enthusiastic appreciation. The chapter on rickets is a masterly little monograph, the chapters on the nervous system, on the kidney, and intestinal tract are especially good; in fact, where there is such a high general standard it is rather an invidious task to single particular articles out for praise. As an instance of the way in which the book is brought up to date, it may be mentioned that there are two descriptions of Von Gierke's disease (hepato nephromegalia glycogenica) with reference to Worcester Drought's review of the literature in 1933. The insulin-glucose treatment in severe diphtheria is also described. There are, of course, as in every work, minor matters where the view-point of the reader will differ from that of the writer, but no one in search of sound progressive views on diseases of children will be likely to go away disappointed after a perusal of these pages. We congratulate editors, contributors, and not least the publishers, upon a worthy achievement.

**The Influence of Heredity on Disease.** By L. S. PENROSE, M.A., M.D. Pp. vii., 78. Illustrated. London: H. K. Lewis & Co. Ltd. 1934. Price 5s.—The author has succeeded in furnishing a concise description of the modern methods employed in the study of human genetics, and in view of the intricate mass of data which have to be dealt with, it is not surprising that special statistical methods have been evolved, with the result that the student who is unfamiliar with mathematical reasoning must find these scientific advances difficult to understand. An instance of the help we can derive from Mendelian analysis is the appreciation of what result to expect from an attempt to eradicate an hereditable feature by controlling propagation in a condition such as mental deficiency. Prevention of breeding would only result in a partial reduction of the incidence, the reason being that if 1 per cent. of the population exhibit a recessive character 18 per cent. must possess it in heterozygous form, and these latter individuals will perpetuate the condition. The author lays stress upon the importance of the numerical analysis of sibships and of the recognition of the environment as a significant factor. This essay should serve as a useful guide to the eugenist as to the most direct lines along which to conduct his researches. We congratulate the author upon the lucid exposition of so complex a subject.

